# Complete mitochondrial genome of the Partridge Shank chicken (Galliformes: Phasianidae)

**DOI:** 10.1080/23802359.2019.1673232

**Published:** 2019-10-01

**Authors:** Min Wang, Ling Wang, Cheng-Qiao Wang, Sheng-Hao Wan, Hu Wang, Jie-Yu Sun, Jian-Qiang Tang, Xiao-Chun Zhang, Hai-Jun Zhang, Jun Li, Shu-Ying Peng

**Affiliations:** Human and Animal Genetics Laboratory, School of Life Science, Huaibei Normal University, Huaibei, Anhui, People’s Republic of China

**Keywords:** Mitochondrial genome, Galliformes, Phasianidae, Partridge Shank chicken, *Gallus gallus domesticus*

## Abstract

Partridge Shank chicken is a valuable broiler breed in China. The first complete mitochondrial DNA (mtDNA) sequence of Partridge Shank chicken had been obtained using PCR amplification, sequencing and assembling. The complete mitochondrial genome was 16,788 bp in length, with the base composition of 30.2% for A, 23.7% for T, 32.5% for C and 13.5% for G. It exhibited the typical mitochondrial structure, including 2 ribosomal RNA genes, 13 protein-coding genes, 22 transfer RNA genes, and a non-coding control region (D-loop region). The phylogenetic tree construced with maximum-likelihood (ML) method based on the complete chicken mitochondrial genomes showed that the 26 chicken breeds could be divided into two groups.

Partridge Shank chicken is one of the important native chicken breed in China. It has the physical characteristics of ‘one wedge,’ ‘two thin,’ and ‘three pockmarked body’. ‘One wedge’ means that the hen has a wedge-like shape body with strong forepart and big and fat posterior. The ‘two thin’ is referred to the thin head and foot. The ‘three pockmarked body’ refers that the color of the hen’s back feather is yellow, brown or blended with both. The chicken possesses the both advantages of growth rate as white-feather broiler and breeding performance just like yellow-feather broiler and shows the characteristics of strong adaptability, rapid growth, and high reproductive performance (Xi et al. 2014; Zhao et al. 2019). Moreover, the meat of Partridge Shank chicken is tender and nutritious.

To investigate the genetic resource, we reported the sequence of the complete mitochondrial genome (mitogenome) of Partridge Shank chicken for the first time. In this study, the Partridge Shank chicken was obtained from local conservation farm in Shizi town, Chuzhou city (N 31°51′–33°13′, E117°09′–119°13′), Anhui Province, China. The whole blood samples were drawn from wing vein and stored at -80 °C in the Human and Animal Genetics Laboratory, College of Life Science, Huaibei Normal University, Huaibei city, Anhui province. Total genomic DNA was extracted from these blood samples by using the TIANamp Genomic DNA Exaction Kit (TIANGEN Biotech Co. Ltd, Beijing, China) according to its instruction manual. The mitogenome was amplified with 20 pairs primers designed according to the mitogenome sequence of Gallus (NC_007236), and PCR products were sequenced by BGI Biotech Co. Ltd (Shanghai, China). Mitogenome sequences were assembled using Seqman Pro software (DNASTAT Inc., Madison, WI) and annotated using MITOS WebServer (http://mitos.bioinf.uni-leipzig.de) (Bernt et al. 2013).

The complete mitogenome of Partridge Shank chicken was 16,788 bp in length (GenBank accession number MN013407). The overall base composition of mitogenome was 30.2% A, 23.7% T, 32.5% C, and 13.5% G and biased A + T (53.9%). It consisted of 37 mitochondrial genes, including 13 protein-coding genes (PCGs), 22 transfer RNAs, 2 rRNA (12S rRNA and 16S rRNA) and a non-coding control region (D-loop region). The arrangement of all genes and transcriptional direction were identical with those of typical Gallus (Desjardins and Morais 1990; Liu et al. 2016; Yan et al. 2016). The mitochondrial genome was very closely arranged with gene overlaps. The total length of gene overlaps was 32 bp in eight different locations (*tRNA^Phe^*-*12S rRNA*, *tRNA^Gln^*-*tRNA^Met^*, *ND2*-*tRNA^Trp^*, *tRNA^Cys^*-*tRNA^Tyr^*, *COI*-*tRNA^Ser^*, *ATP8*-*ATP6*, *ATP6*-*COXIII*, and *ND4L*-*ND4*) with the overlapped size of 1, 1, 2, 1, 9, 10, 1, and 7 bp, respectively. The total length of intergenic spaces was 47 bp in 16 different locations ranging from 1–9 bp, with the largest interval between *tRNA^Leu^*-*ND1* (9 bp). The 13 protein-coding genes of chicken were cytochrome b gene (*Cytb*), cytochrome c oxidase 3 subunit genes (*COI*, *COII* and *COIII*), and NADH oxidoreductase 7 subunit genes (*ND1*, *ND2*, *ND3*, *ND4*, *ND4L*, *ND5* and *ND6*) and 2 subunit genes (*ATPase6* and *ATPase8*). The full length of PCGs was 11,222 bp, accounting for 66.85% of the entire genome. The longest protein-coding gene was *ND5*, which is 1818 bp, and the shortest is the *ATPase8* gene, which is 165 bp. Except for the *ND6* gene and eight tRNA genes (*tRNA^Gln^*, *tRNA^Ala^*, *tRNA^Asn^*, *tRNA^Cys^*, *tRNA^Tyr^*, *tRNA^Ser^*, *tRNA^Pro^* and *tRNA^Glu^*), all mitogenome-genes were encoded on the heavy strand. The PCGs initiation codons were all ATG, except for *COI* that begins with GTG. The termination codons for *COII*, *ATPase6*, *ATPase8*, *ND1*, *ND3*, *ND4L*, *ND5*, *Cytb*, and *ND6* genes were TAA, the termination codon for *ND2* was TAG, the termination codon for *COI* gene was AGG, and the termination codon for *ND4* and *COIII* genes was incomplete termination codon ‘T–’ that was the 5' terminal of the adjacent genes. The incomplete ‘T–’ was supposed to become TAA via posttranscriptional polyadenylation (Anderson et al. 1981). The chicken mitochondrial genome contained 12S rRNA and 16S rRNA. Two ribosomal RNAs were located between the tRNA^Phe^ and tRNA^Leu^ genes, and separated by the tRNA^Val^ gene. The lengths of 12S rRNA and 16S rRNA genes were 976 bp and 1624 bp, respectively. The total A + T content of the 12S rRNA and 16S rRNA gene was 53%, which was slightly lower than the average mitochondrial genome level of 53.9%. The deduced 22 tRNA genes in the chicken mitochondrial genome were distributed among rRNA and protein-coding genes, ranging from 66 to 76 nt. 14 tRNAs were encoded by heavy chains and 8 tRNAs were encoded by light chains. The D-loop region is a 1232 bp long sequence between tRNA^Phe^ and tRNA^Glu^, accounting for 7.3% of the entire gene. Its base A + T content was 60.5%, which was higher than the average mitochondrial genome level of 53.9%. In addition, a cytosine insertion existed in the *ND3* gene. An extra base at position 10,962 was discovered, but reading frame was presumably maintained by translational frameshift of RNA. This phenomenon should be similar to the single nucleotide frameshift insertions in many birds (Mindell et al. 1998).

A maximum-likelihood (ML) tree was constructed based on the complete mitogenomes of 26 chicken breeds using Mega 7.0 (Kimura 1980; Kumar et al. 2016) with 1000 bootstrap replicates in order to elucidate the phylogenetic position of Partridge Shank chicken. The phylogenetic tree showed that the 26 chicken breeds could be clustered two groups using the complete mitochondrial genomes (Figure 1). The 12 chickens including Partridge Shank chicken formed a monophyletic clade, the other chicken breeds formed into another clade. This study will be useful for the phylogenetics of Partridge Shank chicken, and be available as basic data for protection, reproduction and breeding of the poultry.

**Figure 1.**
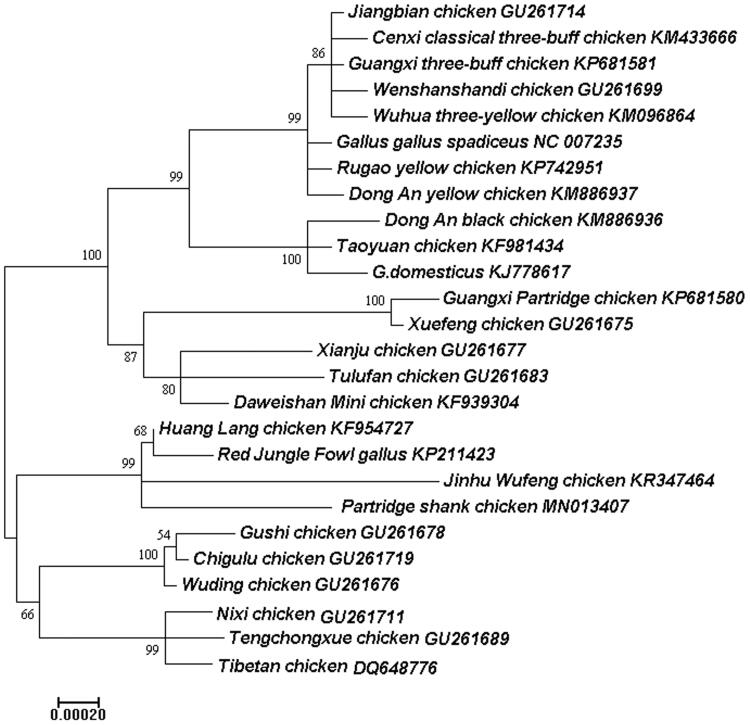
Molecular phylogenetic analysis by ML method based on the complete mitochondrial genomes of 26 chicken breeds. The number at each node indicates the ML bootstrap support values. GenBank accession numbers are given after the species name.
